# Retraction: A negative feedback loop between long noncoding RNA NBAT1 and Sox9 inhibits the malignant progression of gastric cancer cells

**DOI:** 10.1042/BSR-2018-0882_RET

**Published:** 2024-03-01

**Keywords:** angiogenesis, feedback, NBAT1, Sox9

This article is being retracted from *Bioscience Reports* at the request of the Editor-in-Chief and the Editorial Board following receipt of a notification from a reader, alerting the Editorial Board to multiple images within the article that seem to appear in other papers by unrelated authors. Specifically:

- Figure 3A shNC Migration panel which appears as Figure 3 20% SMMC-7721 panel from Peng et al 2017 (DOI: 10.3892/etm.2017.5247)
- Figure 3A shNBAT1 Migration panel which appears as Figure 1F DANCR panel from Mao et al 2017 (DOI: 10.1042/BSR20171070) [retracted], and Figure 2C Con Invasion panel from Xi et al 2017 (PMID: 29218242)
- Figure 3A shNBAT1 Invasion panel which appears as Figure 3 0% SMMC-7221 panel from Peng et al 2017 (DOI: 10.3892/etm.2017.5247), and part of which appears as Figure 2D DANCR Invasion panel from Mao et al 2017 (DOI: 10.1042/BSR20171070) [retracted]
- Figure 4B NBAT1 panel which appears as Figure 2F Con Migration panel from Xi et al 2017 (PMID: 29218242)
- Figure 4E shNBAT1 panel a part of which appears as Figure 2F lncBRM Migration panel from Xi et al 2017 (PMID: 29218242), and part of which appears as Figure 2F Con+siLET Invasion panel from Mao et al 2017 (DOI: 10.1042/BSR20171070) [retracted]

Additionally, the Editorial Board have identified that Figure 3A shNBAT1 Invasion panel also appears as Figure 1E Con panel from Mao et al 2017 (DOI: 10.1042/BSR20171070) [retracted].

The authors have been contacted with regards to the retraction and have not responded to the Journal's queries or the concerns raised. Given the extent of the issues raised, the Editorial Board stand by the decision to retract the article.

